# Genetic analysis of porcine productive and respiratory syndrome virus between 2013 and 2014 in Southern parts of China: identification of several novel strains with amino acid deletions or insertions in nsp2

**DOI:** 10.1186/s12917-019-1906-9

**Published:** 2019-05-24

**Authors:** Shaofeng Hong, Ying Wei, Siyuan Lin, Jiabing Huang, Wei He, Jing Yao, Ying Chen, Ouyang Kang, Weijian Huang, Zuzhang Wei

**Affiliations:** Laboratory of Animal infectious Diseases and Molecular Immunology, College of Animal Science and Technology, Guangxi University, Nanning, 530005 People’s Republic of China

**Keywords:** PRRSV, Genetic analysis, GP5, nsp2, Deletion

## Abstract

**Background:**

Porcine respiratory and reproductive syndrome virus (PRRSV) is one of the most economically significant pathogens in the Chinese swine industry. ORF5 and nsp2 are highly variable regions of the PRRSV genome. Therefore, nsp2 and GP5 are often selected for investigation of variations and phylogenetic analyses for their genetic diversities. Knowledge of the molecular evolution of PRRSV field strains may contribute to the control of PRRS in China.

**Results:**

The results of multiple sequence alignments of GP5 showed that there is 84.5–100% aa identity among the 56 strains in this study. These strains shared 84.5–99.0% aa identity with the prototypical type 2 PRRSV VR-2332 and 56.6–59.2% with strain LV, prototypical type 1 PRRSV. Phylogenetic analysis showed there is considerable diversity among PRRSV ORF5 and the existence of two lineages (5 and 8). Most of the strains were classified into lineage 8 with multiple sub-lineages (3, 4 and 6). Moreover, PRRSV strains with 5 novel patterns of deletions or insertions in the nsp2 region were found.

**Conclusions:**

Phylogenetic analysis based on ORF5 sequences indicated the diversity of PRRSV in southern parts of China and the strains with 30 aa deletion in nsp2 are dominant in the porcine population. Also, new PRRSV strains with different patterns of deletions or insertions in nsp2 are emerging. The data presented here constitute a useful basis for further epidemiological studies regarding the heterogeneity of PRRSV strains in China and provide a basis for the prevention of PRRS in southern parts of China.

**Electronic supplementary material:**

The online version of this article (10.1186/s12917-019-1906-9) contains supplementary material, which is available to authorized users.

## Background

Porcine reproductive and respiratory syndrome virus (PRRSV) is acknowledged as one of the most economically important diseases for the swine industry worldwide [1]. PRRSV, the etiological agent of porcine reproductive and respiratory syndrome (PRRS), is a single-stranded, enveloped, RNA virus. The PRRSV genome consists of approximately15.4 kb and contains a 5′-untranslated region (UTR), open reading frames (ORFs), a 3′-UTR and a 3′-poly(A) tail. The 5′ two-thirds of the genome encodes polyproteins that are processed by viral protease to 14 nonstructural proteins (nsps) [2]. The 3′ one-third region of genome encodes the structural proteins that are translated from a 3′–5′ co-terminal, nested set of subgenomic mRNAs. In addition to the three major structural proteins, GP5, M and N, the genome of PRRSV encodes minor structural proteins, GP2, 2b, GP3, GP4 and ORF5a [3–5].

It has been shown that PRRSV is continuously evolving through point mutations and genome recombination, which can lead to some new emerging antigenic variant strains [6]. According to the genetic diversity, PRRSV has been classified as two separate species: type 1 (European) PRRSV and type 2 (North American) PRRSV. The two genotypes share about 60% identity at the nucleotide level [7]. GP5 is highly variable and contains important immunological domains associated with viral neutralization [8]. Nsp2 is the most variable region of PRRSV genome and substitutions, deletions and insertions have been observed in the nsp2 coding region [9, 10]. Therefore, nsp2 and GP5 are often selected for investigation of variations and phylogenetic analyses for their genetic diversities. The genetically extensive variation of PRRSV is likely to pose a major obstacle for the effective control of the most economically significant disease that affects the swine industry [11].

PRRS outbreaks were documented in an intensive pig farm in China at the end of 1995, and it has become one of the most important swine diseases in the Chinese swine industry. In 2006, a large outbreak of porcine high fever syndrome (PHFD), caused by a highly pathogenic PRRSV (HP-PRRSV), emerged in China and affected over 20 million pigs with about 400,000 fatal cases [12]. The disease was subsequently confirmed in southeastern Asian countries including Vietnam, Laos and the Philippines, and this has caused disastrous damage to the swine industry [13, 14]. Whole-genome analysis of the isolated viruses revealed that these PRRSV strains could be grouped into genotype 2 and were highly homologous to a Chinese isolate HB-1 (96.5% nucleotide identity) [15]. It was furthermore observed that these emerging strains contained a single amino acid deletion at position 481 and a 29-amino acid deletion from position 532 to 560 in nsp2 [12, 16]. The study with regards to the origin of the HP-PRRSV did not find recombination or large fragment replacement, which suggests that all HP-PRRSVs originated from the same Chinese ancestor by gradual evolution [15].

Guangxi is one of the biggest breeding regions in the southern parts of China. The genotype prevalence of PRRSV in South China is not currently known. The aim of this study is to investigate the genetic variation of PRRSV using strains isolated in 2013–2014 from pigs that exhibited symptoms of the disease.

## Results

### Prevalence of PRRSV in Guangxi Province, China from 2013 to 2014

Of the 475 filed samples collected from clinically diseased pigs found between 2013 and 2014 in Guangxi Province of China, 133 samples (28%) were positive for PRRSV, as determined by specific PCR. These results indicated that PRRSV was widely distributed among swine populations in the southern parts of China.

### Sequence analyses of the ORF5 gene and nsp2 hypervariable regions of PRRSV

**Fig. 1:**
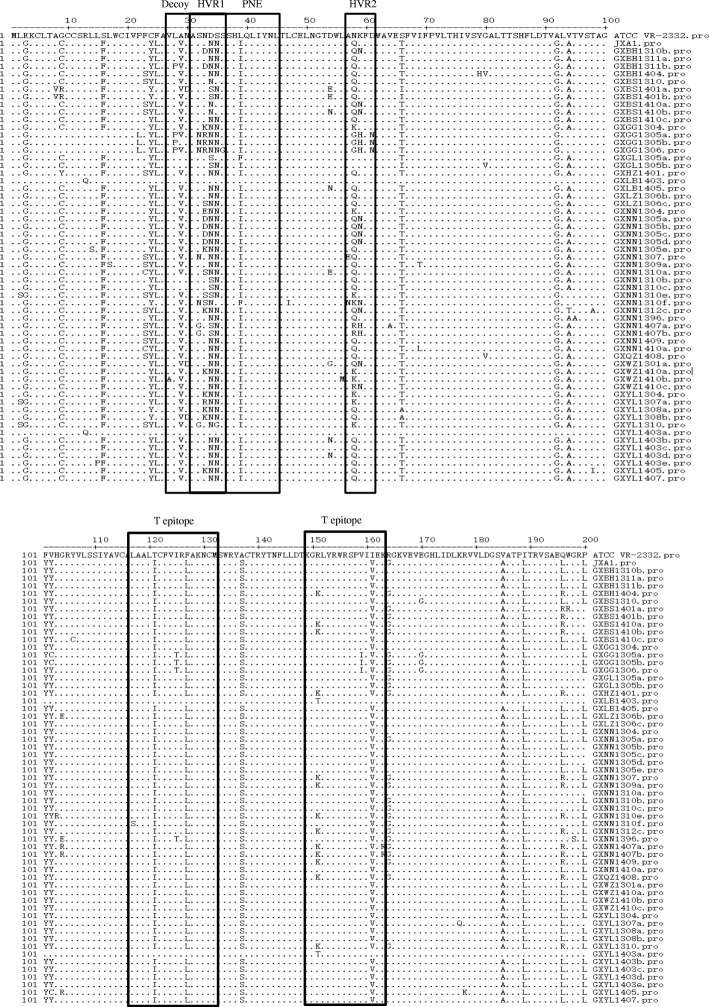
The alignment of GP5 of PRRSV. A multiple alignment of PRRSV GP5 was performed by Clustal W. The sequence of VR-2332 is shown at the top; the residues conserved with it are hidden and substitutions are indicated by the amino acid letter codes. The functional domains are shown within boxes. HRV: highly variable regions, DCE: decoy epitope, PNE: primary neutralizing epitope

One hundred and thirty-three PRRSV positive samples were used for ORF5 gene and nsp2 HVR amplification. Fifty-six ORF5 sequences and 35 nsp2 HVR sequences were selected for sequencing and analysis (Table 1). To investigate the amino acid difference among PRRSV strains, the GP5 amino acid sequences of 56 PRRSV strains were aligned, together with some North American genotype strains and those from China and other countries. The results showed that all 56 strains encoded a GP5 protein of 200 amino acid residues, but substitutions were extensive (Fig. 1). Sequence alignments revealed that there was 84.5–100% amino acid identity between the 56 Guangxi PRRSV strains and shared 84.5–99.0% amino acid identity with the prototypical type 2 PRRSV VR-2332, 87.0–99.0% with JX-A1, 89.5–92.5% with CH-1a and 56.6–59.2% with prototypical type 1 PRRSV strain LV (Additional file 1: Table S1).

**Table 1:** Geographic origin and amplified sequence size of target genes from clinical samples in this study

No	Name of strain	Collection date	Area	ORF5(bp)/accession number	NSP2(bp)/accession number
1	GXBH1310b	2013.10	Baihai	603/MG604994	NA
2	GXBH1311a	2013.11	Baihai	603/MG604995	NA
3	GXBH1311b	2013.11	Baihai	603/MG605048	NA
4	GXBH1404	2014.04	Baihai	603/MG605047	1322/MG604959
5	GXBS1310	2013.10	Baise	603/MG605046	1682 /MG604960
6	GXBS1401a	2014.01	Baise	603/MG605045	1679 /MG604961
7	GXBS1410a	2014.10	Baise	603/MG605043	1682 /MG604962
8	GXBS1410b	2014.10	Baise	603/MG605042	1682 / MG604963
9	GXBS1410c	2014.10	Baise	603/MG605041	1682 / MG604964
10	GXGG1304	2013.04	Guigang	603/MG605040	NA
11	GXGG1305a	2013.05	Guigang	603/MG605039	NA
12	GXGG1305b	2013.05	Guigang	603/MG605038	NA
13	GXGG1306	2013.06	Guigang	603/MG605037	1682 / MG604965
14	GXGL1305a	2013.05	Guilin	603/MG605036	NA
15	GXGL1305b	2013.05	Guilin	603/MG605035	NA
16	GXHZ1401	2014.01	Hezhou	603/MG604999	1322/MG604966
17	GXLB1403	2014.03	Laibin	603/MG605034	NA
18	GXLZ1405	2014.05	Laibin	603/MG605033	1682/MG604968
19	GXLZ1306b	2013.06	Liuzhou	603/MG605032	1682/MG604967
20	GXLZ1306c	2013.06	Liuzhou	603/MG605031	NA
21	GXNN1304	2013.04	Nanning	603/MG605030	1682/MG604969
22	GXNN1305a	2013.05	Nanning	603/MG605029	1682/MG604970
23	GXNN1305b	2013.05	Nanning	603/MG605028	1682/MG604971
24	GXNN1305c	2013.05	Nanning	603/MG605027	1682/MG604972
25	GXNN1305d	2013.05	Nanning	603/MG605026	NA
26	GXNN1305e	2013.05	Nanning	603/MG605025	1682/MG604973
27	GXNN1307	2013.07	Nanning	603/MG605024	NA
28	GXNN1309a	2013.09	Nanning	603/MG605023	NA
29	GXNN1310a	2013.10	Nanning	603/MG605022	NA
30	GXNN1310b	2013.10	Nanning	603/MG605021	NA
31	GXNN1310c	2013.10	Nanning	603/MG605020	NA
33	GXNN1310f	2013.10	Nanning	603/ MG605019	NA
34	GXNN1312c	2013.12	Nanning	603/MG605018	1682/MG604974
35	GXNN1396	2013.09	Nanning	603/MG605049	1626/MG604975
36	GXNN1407a	2014.07	Nanning	603/MG604998	1685/MG604976
37	GXNN1407b	2014.07	Nanning	603/MG604997	1682/MG604977
38	GXNN1409	2014.09	Nanning	603/MG604996	1682/MG604978
39	GXNN1410a	2014.10	Nanning	603/MG605017	1682/MG604979
40	GXQZ1408	2014.08	Qinzhou	603/MG605016	NA
41	GXWZ1301a	2013.01	Wuzhou	603/MG605015	1682/MG604981
42	GXWZ1410a	2014.10	Wuzhou	603/MG605014	NA
43	GXWZ1410b	2014.10	Wuzhou	603/MG605013	1682/MG604982
44	GXWZ1410c	2014.10	Wuzhou	603/MG605012	NA
45	GXYL1304	2013.04	Yulin	603/MG605011	1682/MG604983
46	GXYL1307a	2013.07	Yulin	603/MG605010	1682/MG604984
47	GXYL1308a	2013.08	Yulin	603/MG605009	NA
48	GXYL1308b	2013.08	Yulin	603MG605008/	1682/MG604985
49	GXYL1310	2013.10	Yulin	603/MG605007	1322/MG604986
50	GXYL1403a	2014.03	Yulin	603/MG605006	1722/MG604987
51	GXYL1403b	2014.03	Yulin	603/MG605005	1682/MG604988
52	GXYL1403c	2014.03	Yulin	603/MG605004	NA
53	GXYL1403d	2014.03	Yulin	603/MG605003	1682/MG604989
54	GXYL1403e	2014.03	Yulin	603/MG605002	1400/MG604990
55	GXYL1405	2014.05	Yulin	603/MG605001	1682/MG604992
56	GXYL1407	2014.07	Yulin	603/MG605000	1682/MG604993

*NA* not amplified

To investigate variation in the deduced amino acid sequences of ORF5 gene products, the amino acid sequences of 56 PRRSV strains including some representative strains were aligned. As shown in Fig. 1, critical amino acid variations in some motifs and regions such as the peptide signal, HRV, the decoy epitope (DCE), the primary neutralizing epitope (PNE) and T epitopes were found in GP5 of these strains. Decoy epitopes of VR-2332 is ^27^VLAN^30^ and of JXA1 is ^27^VLVN^30^. Strains GXLB1403, GXGL1305a/b and GXNN1310b/c contained the same aa in their decoy epitopes (^27^VLAN^30^) as those of VR2332. Specific substitution at position 27 (^27^V → ^27^A) was found in strain GXWZ1401b, while strains GXGG1305a, GXGG1306 and GXBH1311b contained variations at positions 28 (^28^L → ^28^P) and strains GXBS1401a, GXWZ1301a and GXYL1308b have a specific substitution at position 30 (^30^N → ^30^D) in the decoy epitope compared to those of VR2332 and JXA1. The other strains have the same aa at the decoy epitope as those of JXA1. Great diversities in HVR1 and HVR2 were found at amino acid positions 32–37 and 57–62, respectively. Specifically, substitutions (S^32^ → N^32^, S^32^ → G^32^, N^34^ → S^34^, N^35^ → G^35^ and S^36^ → G^36^) in HVR1 of some strains, resulted in loss or gain of the N-linked glycosylation site at specific position in GP5 (Table 2). Amino acid substitutions in the primary neutralizing epitope were also found. Strains GXLB1403, GXGL1305a/b and GXNN1310b/c contained the same aa in the primary neutralizing epitope (^37^SHLQLIYNL^45^) as those of VR2332. All PRRSV strains in sub-genotypes 3, representative of JXA1, contained an amino acid mutation (^39^L → ^39^I) when compared with VR-2332, while strains GXLB1403 and GXYL1403a contain variations at positions 39 (^39^L → ^39^F). Substitutions (N^58^ → Q/G/K/R^58^, K^59^ → H/N^59^ and D^60^ → N^60^) in HVR2 were observed in some strains compared with VR2332. In T cell epitopes, strains GXGG1305a, GXGG1305b and GXGG1306 carried substitutions at positions 126(I^126^ → T^126^). Some strains carried substitutions at position at 151(R^151^ → K^151^) (Fig. 1).

**Table 2:** The potential N-glycosylation sites in GP5 of different strains in this study

Name of isolates	The number of N-glycosylation sites	The position of N-glycosylation sites
GXNN1310f, GXYL1310	3	N34, N44, N51
GXGG1306, GXNN1407a, GXBS1401a	3	N35, N44, N51
GXGG1305a/b, GXNN1307, GXWZ1301a, GXYL1307a, GXYL1308b	4	N34, N35, N44, N51
GXLB1403, GXYL1403a, GXGL1305a	4	N30, N33, N44, N51
GXGL1305b, GXNN1310b/c/e, GX1407b, GXBS1401b	4	N30, N35, N44, N51
GXBS1310, GXBS1410a/b	5	N30, N33, N34, N44, N51
GX1409, GXBH1310b, GXBH1311a/b, GXBH1404, GXBS1410c, GXGG1304, GXYL1304, GXLB1405, GXYL1430b/d, GXLZ1306b/c, GXNN1304, GXNN1305a/b/c/d/e, GXNN1309a, GXNN1310a, GXNN1312c, GXNN1396, GXNN1410a, GXQZ1408, GXWZ1410a/b/c, GXYL1308a, GXYL1403c/e, GXYL1407, GXYL1405, GXHZ1401	5	N30, N34, N35, N44, N51

### Identification of several novel strains with amino acids deletions or insertions in nsp2

The nsp2 gene has the highest genetic diversity in the genomes of PRRSV field strains and also was used as an epidemiological genetic marker. To investigate the amino acid differences among PRRSV strains, a predicted 1862-bp DNA fragment containing nsp2 HVR from 35 positive samples was amplified, cloned and sequenced. As shown in Table 1, the amplified nsp2 HVR exhibited various lengths. Compared to strains VR-2332, 1 of 35 nsp2 sequences was 1722 nucleotides in length which is the same as that of VR-2332. 29 out of 35 nsp2 HV region sequences had the same length of 1682 nucleotides, containing the same 30-aa deletion as JXA1 and other HP-PRRSV strains, suggesting that strains with a 30 aa deletion in nsp2 is the dominating strain circulating in the southern parts of China.

**Fig. 2:**
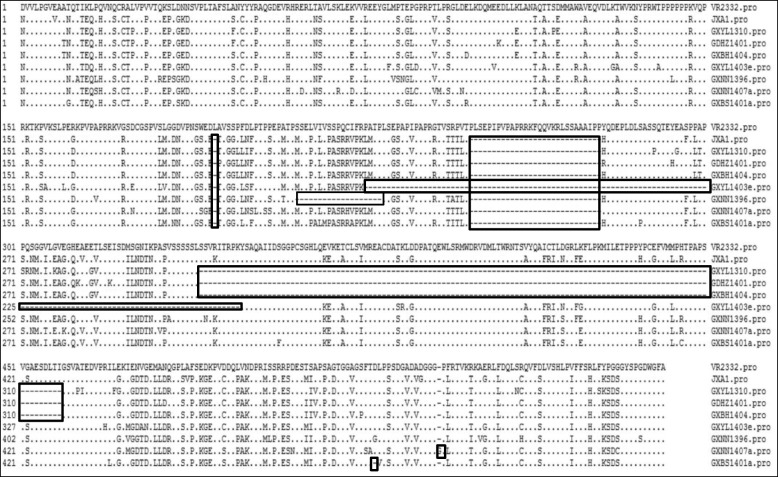
Identification of PRRSV strains with amino acid deletions or insertions in nsp2. A multiple alignment of PRRSV NSP2 HV was performed by Clustal W. The sequence of VR-2332 is shown at the top; the residues conserved with it are hidden. The deleted or inserted amino acids are labeled with boxes

Strains GXYL1310, GDHZ1401 and GXBH1404 had the same length of 1322 nucleotides and were found to contain the same 30-aa deletion as JXA1. They also have an extra continuous 120 aa deletion in nsp2. Strains GXBS1401a, GXNN1396 and GXYL1403e contained a discontinuous 31, 49 and 123 aa deletion in their HVR, respectively, compared with strainVR-2332. We also found that one isolated strain (GXNN1407a) contained a 30 aa deletion and 1 aa insertion compared with VR-2332 and JXA1 (Fig. 2). Pairwise comparisons revealed that 86.4–100% nucleotide identity and 84.5–100% amino acid identity between the 35 Guangxi PRRSV strains and shared 65.9%*~* 99.2% amino acid identity with the prototypical type 2 PRRSV VR-2332, 87.0–99.0% with JX-A1, 89.5–92.5% with CH-1a and 56.6–59.2% with strain LV of the European type. The data suggested that nsp2 is highly variable and novel HP-PRRSV strains with aa deletions and insertions in the nsp2 are emerging.

### Phylogenetic analyses of the ORF5 gene and nsp2 hypervariable region of PRRSV

**Fig. 3:**
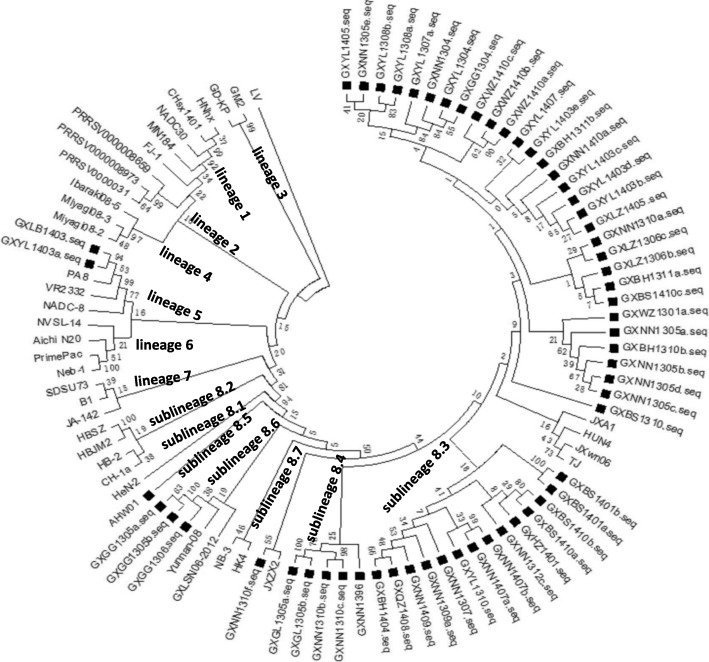
Phylogenetic tree based on a comparison of 95 complete PRRSV ORF5 sequences, including the 56 sequences from this study and 39 ORF5 sequences from PRRSV strains originating from China and other countries. The tree was constructed using the neighbor-joining algorithm based on the p-distance model. The numbers indicate percentage bootstrap values calculated from 1000 bootstrap replicates*.* The PRRSV ORF5 sequences collected in this study are marked with a black square. The ORF5 sequences from prototype viruses of PRRSV are marked with a black square

To gain a better understanding of the genetic relationship, the phylogenetic analysis based on deduced amino acid sequences of ORF5 gene products was conducted by using the 56 ORF5 sequences obtained in this study together with 39 downloaded referenced PRRSV sequences (Table 3). As shown in Fig. 3, the results showed that the PRRSV strains in this study could be divided into 2 different subgroups. Among the 56 GP5 sequences, two strains (GXBL1403 and GXYL1403a) belonged to lineage 5, as represented by VR-2332. Fifty-four strains belonged to lineage 8, with six strains being classified as sub-lineage 8.4 and three strains being classified as sub-lineage 8.6. The other 45 strains formed a large cluster being classified as sub-lineage 8.3 with the representative strains being JXA1, JXwn06 and HUN4.

**Table 3:** The information of reference strains used in this study

No.	Virus strain	Accession no.	Country	Lineage
1	NADC30	JN654459	American	1
2	MN184	EF442777	American	1
3	CHsx1401	KP861625	China	1
4	HNhx	KX766379	China	1
5	PRRSV0000008659	EU758687	American	2
6	PRRSV0000008973	EU758940	American	2
7	PRRSV0000031	DQ474791	American	2
8	FJ-1	AY881994	China	3
9	GD-KP	KU978619	China	3
10	GM2	JN662424	China	3
11	Ibaraki08–5	AB546113	Japan	4
12	Miyagi08–2	AB546105	Japan	4
13	Miyagi08–3	AB546106	Japan	4
14	VR-23332	AY150564	American	5
15	NADC-8	AF396833	American	5
16	PA8	AH006184	Canada	5
17	NVSL-14	AF396839	American	6
18	Aichi N20	AB175715	Japan	7
19	Neb-1	EU755263	American	7
20	PrimePac	AF066384	American	7
21	CH-1a	AY032626	China	8.1
22	HH08	JX679179	China	8.1
23	HBJM2	EU399826	China	8.2
24	HBSZ	EU399825	China	8.2
25	JXA1	EF112445	China	8.3
26	JXwn06	EF641008	China	8.3
27	TJ	EU860248	China	8.3
28	GXLSN06–2012	KC618172	China	8.4
29	JXZX2	EU399849	China	8.4
30	AHW01	EU399828	China	8.5
31	HeN-2	FJ237419	China	8.5
32	JXZX2	HQ832215	China	8.6
33	Yunnan-08	EU819086	China	8.6
34	HK1	KF287132	China	8.7
35	HK4	KF287134	China	8.7
36	JA-142	AF396842	American	9
37	SDSU73	AY656993	American	9
38	B1	AY318773	American	9
39	LV	M96262	Netherlands

## Discussion

Since its emergence in China at the end of 1995, PRRSV has spread widely in all areas of China and is continuously evolving. This has led to the emergence of some new antigenic variant strains [17]. In 2006, a large outbreak of porcine high fever syndrome (PHFD), caused by a highly pathogenic form of PRRSV, emerged in China and Southeast Asian countries and caused major economic losses for swine farming [12, 15, 16] . In our previous study, 133 of 475 samples (28%) were positive for PRRSV, indicating that PRRSV is widely distributed among swine populations in southern parts of China**.** Fifty-six ORF5 sequences and 35 nsp2 HVR sequences were selected for investigation of variations and phylogenetic analyses for their genetic diversities. Sequences alignments of GP5 and nsp2 showed that there was extensive genetic variability between them (84.5–100% amino acid identity) or with the representative strain, VR-2332 (84.5–99.0% amino acid identity). GP5 based phylogenetic trees showed all these strains belonged to the type 2 PRRSV which are scattered in 2 lineages (lineages 5 and 8). But most of the strains belonged to a large cluster in sub-lineage 8.3 with the representative HP-PRRSV strains being JXA1, JXwn06 and HUN4. This is consistent with other studies showing that the dominant PRRSV seen in Guangdong Province, which is also located in Southern China, was HP-PRRSV between 2007 and 2014 [18, 19]. The sub-lineage 8.3 PRRSV was also the predominant virus at the country-wide scale in the subsequent years since 2007 [17, 20, 21].

As a transmembrane protein, GP5 possesses two to four potential N-linked glycosylation sites that are located in a small ectodomain [22]. The N-linked glycosylation of GP5 have been shown to be involved in diverse functions such as the proper folding protein, receptor binding, virus infectivity and induction of immune response [8, 23–25]. The amino acids in the proximal region of the ectodomain of GP5 are highly variable. In this study, we showed that substitutions at each consensus sequence of N-linked glycosylation site, N-X-T/S, in GP5 of some strains, resulted in loss or gain of N-linked glycosylation sites at specific positions in GP5. The N44 and N51-linked glycosylation sites were well conserved. The DCE upstream of the PNE was speculated to elicit a great abundance of the non-neutralized antibodies against GP5 and delay the production of neutralizing antibodies stimulated by PNE [8]. The alignment of GP5 showed that variations in DCE were observed and key aa substitutions in PNE were found among the strains in this study. As a result, a different number of N-glycosylation sites among the strains and the key aa variations in DCE and PNE might allow these field strains to escape neutralization by the antibodies induced by current vaccines.

The results of alignment and phylogenetic tree studies based on GP5 and HV of nsp2 also showed that a great number of emergences of PRRSV might be related to the extensive use of the attenuated modified live virus (MLV) PRRS vaccine in China. Three strains, GXYL1310, GDHZ1401 and GXBH1404, which have a specific 120 aa deletion in nsp2 were identified. It was suggested that there is the possibility that these three strains were derived directly from the widely used commercial vaccine strain, TJM, which is characterized by a 120 aa deletion in nsp2 and derived originally from the TJ strain by serial passage in MARC-145 cells of up to 92 times [26]. VR2332-derived MLV strains were also found. Two strains were clustered into this minor branch shared a high identity with MLV vaccine and its parent virus VR-2332, with amino acid similarities of 99.2 and 99.7%, respectively. Several studies showed that some prevalent PRRSV strains may be related to the reversion of commercial MLVs and the recombination between the vaccine virus and field viruses [27–31]. It has been suggested that more attention should be paid to MLV-like strains which have undergone evolutionary changes and have since circulated widely in the field.

The nsp2 of PRRSV is a highly heterogeneous protein. Remarkably, natural deletions and insertions have continued to occur in the HV of nsp2, and these have led to genome size differences among PRRSV strains [9, 32, 33]. Type 2 PRRSV with 1–150 aa deletions and 1–36 aa insertions in the nsp2 coding region has been identified in USA, China, Japan, Denmark and Thailand [9, 10, 33, 34]. In this study, most of the strains have a discontinuous 30 aa deletion, suggesting that the strain with a 30 aa deletion in nsp2 is the dominant virus prevalent in the southern parts of China. It is noted that several novel strains (GXNN1396, GXYL1403e, GXBS1401a and GXNN1407a) with additional aa deletions or insertions are also found, suggesting that strains with other types of aa deletions in nsp2 may have also been prevalent in this region. GXNN1396 has a discontinuous 30 aa deletion and a continuous 19 aa deletion at position 499–517 in nsp2 compared to VR-2332. Similar 19 aa deletions in nsp2 were also observed in a Japanese PRRSV strain, Jyc, and several USA PRRSV strains (MN184, NADC30 and NADC31), which have 19 aa deletions at position 495–513. GXYL1403e had a novel discontinuous 124 aa deletion at positions 481 and 496–619 in the nsp2-coding region in contrast to the VR2332 reference strain. Similar aa deletions at this region were also observed in a Chinese PRRSV strain, Em2007, which has a 68 aa deletion at position 499–566. Compared to JXA1, strains GXBS1401a and GXNN1407a have one aa deletion at position 816 and one aa insertion at position 830, respectively. One aa insertion in nsp2 was also identified in a Japanese strain, Jtg, which have one aa insertion at position 554 (Additional file 2: Table S2). The emergence of HP-PRRSV strains with 30 aa deletions in the nsp2 was once speculated to be related to its virulence. However, the following study showed that the discontinuous deletion of 30 amino acids in nsp2 was not related to the virulence of the emerging HP- PRRSV [35]. But recent studies showed that PRRSV strains with deletions in the nsp2 were more likely to be pathogenic [33, 35, 36]. Overall, the mechanism underlying spontaneous deletions in nsp2 during viral passages in vivo and their effect on viral replication and pathogenicity remains unclear.

Nsp2 is also a highly immunogenic protein. It has been shown that nsp2 contains several putative B-cell and T-cell epitopes. Antibodies against nsp2 were generated as early as 1 week after PRRSV infection [37, 38]. Most of these epitopes were found to be mapped to the HV of nsp2 which usually occur by substitutions, natural deletions and insertions. It has been shown that a natural deletion or an engineered deletion in nsp2 of PRRSV plays an important role in modulating the induction of inflammatory cytokines in vitro [36, 39, 40]. The biological and immunological characteristics of the strains with specific aa deletions in nsp2 remain topics for further studies.

## Conclusions

In this study, we showed PRRSV is widely distributed among swine populations in the southern parts of China. GP5 based phylogenetic trees and sequence alignments showed that extensive genetic variability exists compared with the representative stains and the PRRSV strains with 30 amino acid deletions in nsp2 and these are dominant in the porcine population. In addition, more and more PRRSV strains with different patterns of deletions or insertions in nsp2 are emerging. This study expands the existing knowledge of the genetic diversity and evolution of PRRSV in southern parts of China and can potentially help to better control the spread of PRRSV.

## Methods

### Sample collection, viral RNA extraction and PRRSV detection

Field samples *(n* = 475*)* (sera, lungs, lymph nodes and spleens) from clinically diseased pigs between 2013 and 2014 in different regions of Guangxi, China were submitted to the Laboratory of Animal infectious Diseases and Molecular Immunology, Guangxi University, Nanning for PRRSV testing. A summary of the samples studied is presented in Table 1. Total RNA was extracted using TRizol reagent (Invitrogen, Grand Island, NY, USA) according to the manufacturer’s instructions and then used for synthesis of cDNA with random hexamers (Fermentas, Glen Burnie, MD, USA). All the samples were screened for PRRSV by PCR using the forward and reverse primers, (5′*-*AAGCTGTTAAACAGGGAGTGG-3′) and (5′*-*CCAAAGAATACCAGCCCATCA-3′), respectively*.* Thermal cycling conditions were 95 °C for 3 min, followed by 35 cycles of 95 °C for 1 min, 59 °C for 40s, 72 °C for 1 min, and a final elongation step at 72 °C for 10 min. Finally, the PCR products were analyzed on 1.0% agarose gel electrophoresis ultraviolet imaging. Positive samples were determined by the presence of 443 bp amplified products.

### Cells and virus

MARC-145 cells were grown at 37°Cin minimum essential medium (MEM) supplemented with 10% fetal bovine serum (FBS). Sera or supernatants of tissue homogenates from PRRSV-positive samples were used to inoculate the MARC-145 cells for PRRSV isolation.

### Amplification of ORF5 and nsp2 hypervariable region and sequence determination

PRRSV positive samples were used for amplification of complete ORF5 and nsp2 hypervariable regions (HVR). The forward (5′-AGGTGGGCAACCGTTTTA-3′) and reverse primers (5′-CATCACTGGCGTGTAGGTAAT-3′) were used for amplification of the complete ORF5. PCR reaction conditions were 95 °C for 3 min, followed by 30 cycles of 95 °C for 1 min, 59 °C for 1 min, 72 °C for 1.5 min, and a final elongation step at 72 °C for 10 min. The forward (5′-AATGTTGTTCTTCCTGGGGTTGAG-3′) and reverse primers (5′-AAGCTGCAAAACCCCAATCACCCG-3′) were used for amplification of the nsp2 HVR. PCR reaction condition was 95 °C for 3 min, followed by 30 cycles of 95 °C for 40 s, 57 °C for 40 s, 72 °C for 2 min, and a final elongation step at 72 °C for 10 min. The PCR products were purified with an E.Z.N.A.TM Gel Extraction Kit (OMEGA, USA) and cloned into *pBST-II* vector (TIANGEN Inc., Beijing, China). Positive clones were sequenced in both directions using universal primers T7 and SP6 promoter-specific primers.

### Amino acid mutation analysis of Nsp2 HV and ORF5

To further characterize the amino acid mutation in Nsp2 HV and GP5, differences of the amino acid sequences derived from ORF5 gene and nsp2 HVR of these strains and other representative strains from China and other countries were analyzed and aligned using the MegAlign program (version 5.01) of the DNASTAR package. (DNASTAR Inc., Madison, WI, USA).

### Phylogenetic tree analysis

The multiple sequence alignment of the nucleotide sequences of ORF5 or nsp2 HVR were performed by using the Clustal W method in MEGA5.2. MEGA version 5.2 with the p-distance model was used to evaluate phylogenetic relationships by the neighbor-joining method with 1000 bootstrap replicates. The sequences obtained in this study were submitted to the GenBank database under the accession numbers (MG604994 - MG605049 for ORF5 and MG604959 - MG604993 for nsp2) and the reference strains from China and other countries (lineages 1 to 9) used in this study are listed in Table 2. The classification of lineages and sub-lineages was according to their description in recent studies [41, 42].

## Additional files

Additional file 1: **Table S1.** Comparison of the GP5 sequences of the different PRRSV strains examined in this study. (DOCX 14 kb)

Additional file 2: **Table S2.** The positions and sizes of aa insertions and deletions in nsp2 of PRRSV strains compared to VR2332. (DOCX 26 kb)

## Abbreviations

DCE: decoy epitope
HP: Highly pathogenic
HVR: hypervariable region
nsp: nonstructural proteins
ORF: Open reading frame
PRRS: Porcine reproductive and respiratory syndrome
PRRSV: Porcine reproductive and respiratory syndrome virus
