# Supplementary material for: Genetic analysis of porcine productive and respiratory syndrome virus between 2013 and 2014 in Southern parts of China: identification of several novel strains with amino acid deletions or insertions in nsp2
Source: BMC Vet Res. 2019 May 24;15:171. doi: 10.1186/s12917-019-1906-9 (PMC6534915; doi:10.1186/s12917-019-1906-9)
Supplement: Supplementary file 1 — Table S1. Comparison of the GP5 sequences of the different PRRSV strains examined in this study. (DOCX 14 kb) [file 12917_2019_1906_MOESM1_ESM.docx]

|  | U87392  (ATCC-VR-2332) | AY032626  (CH-1a) | EF112445  (JXA1) | M96262  (LV) | PRRSVs  (56 strains) |
| --- | --- | --- | --- | --- | --- |
| U87392  (ATCC-VR-2332) | 100.0 | 92.0 | 89.2 | 63.7 | 84.5~99.0 |
| AY032626  (CH-1a) |  | 100.0 | 95.4 | 63.8 | 89.5~92.5 |
| EF112445  (JXA1) |  |  | 100.0 | 63.2 | 87.0~99.0 |
| M96262  (LV) |  |  |  | 100.0 | 56.6~59.2 |
| PRRSVs  （56strains） |  |  |  |  | 84.5~100.0 |

Supplementary Table 1. Comparison of the GP5 sequences of the different PRRSV strains examined in this study.
