# Supplementary material for: Genetic analysis of porcine productive and respiratory syndrome virus between 2013 and 2014 in Southern parts of China: identification of several novel strains with amino acid deletions or insertions in nsp2
Source: BMC Vet Res. 2019 May 24;15:171. doi: 10.1186/s12917-019-1906-9 (PMC6534915; doi:10.1186/s12917-019-1906-9)
Supplement: Supplementary file 2 — Table S2. The positions and sizes of aa insertions and deletions in nsp2 of PRRSV strains compared to VR2332. (DOCX 26 kb) [file 12917_2019_1906_MOESM2_ESM.docx]

Supplementary Table 1：The positions and sizes of aa insertions and deletions in nsp2 of PRRSV strains compared to VR2332

| Strain Country | | Deletion (and insertion) | | Accession no |
| --- | --- | --- | --- | --- |
|  |  | aa size | Range of aa positions in nsp2 |  |
| EDRD-1 | Japan | 39  (36) | 495–533  (813–848) | AB288356 |
| Aomori 93-1 | Japan | 1 | 814 | AB288114 |
| Jtg1 | Japan | (1)  31 | (554)  784–814 | AB288117 |
| Jnt1 | Japan | 126 | 304–429 | AB288117 |
| Jyt3 | Japan | 1  (7) | 555 to 556  (801–807) | AB288123 |
| Jsa1 | Japan | 6 | 778–783 | AB288128 |
| Jos1 | Japan | 3 | 594–596 | AB288138 |
| Jyc1 | Japan | 1  19 | 469  495–513 | AB288133 |
| BJ-4 | China | 1 | 694 | AF331831 |
| 19407B | Denmark | 4 | 794–797 | AF303356 |
| P129 | USA | 6 | 505–510 | AF494042 |
| HB-2 | China | 12 | 469–480 | AY262352 |
| JXA1 | China | 1  29 | 481  532–560 | EF112445 |
| MN184A | USA | 111  1  19 | 323–433  485  495–513 | DQ176019 |
| SP | USA | (36) | (813–848) | AF184212 |
| 07NP2 | Thailand | 38  9 | 328-365  466-474 |  |
| 8NP46 | Thailand | 97  1  1 | 333-429  471  513 |  |
| 8NP59 | Thailand | 127  1 | 303-429  471 |  |
| 131101-GD-SHC | China | 1  12  29 | 481  499-510  532–560 | KR612196 |
| SCwhn12DZ | China | 1  29  3 | 481  533-561  594-596 | KT030926 |
| SCwhn13ZY | China | 29  9 | 533-561  573-581 | KT030933 |

| Strain Country | | Deletion (and insertion) | | Accession no |
| --- | --- | --- | --- | --- |
|  |  | aa size | Range of aa positions in nsp2 |  |
| SCmy15 | China | 29  12 | 533-561  577-588 | KY619997 |
| SCcd15-3 | China | 25  29 | 475-499  533-561 | KY620002 |
| FJW05 | China | 30  29 | 470-499  533-561 | KP860911 |
| FJSD | China | 3 | 594-596 | KY444739 |
| FJYR | China | 48  29 | 471-518  533-561 | KT804696 |
| GDQY2 | China | 36  29 | 481-506  533-561 | GU454850.1 |
| XJu-1 | China | 1  29  120 | 481  533-561  628-747 | KF815525 |
| LNWK96 | China | 100 | 328-427 | MG860516 |
| GZ1101 | China | 3 | 593-595 | KF771273 |
| SD0901 | China | 1  1  29 | 468  482  533-561 | JN256115 |
| C1 | China | 1  3 | 503  593-595 | KM433675 |
| Em2007 | China | 68 | 499-566 | EU262603 |
| CG | China | 36  29 | 468–503  533–561 | EU864231 |
| CA-2 | Korea | 111  1  19 | 323–433  482 to 483  495–513 | KF555450 |
| NADC30 | USA | 111  1  19 | 323–433  485  495–513 to 504–522 | JN654459 |
| NADC31 | USA | 111  5  1  19  12 | 323–433  477-481-  485  501-519  582-593 | JN660150 |
| DK-2010-10-1-2 | Denmark | 19  3 | 498-516  593-595 | KC862579 |
| GXHZ1401 | China | 1  29  120 | 481  538-566  628-747 | MG604966 |
| GDYL1310 | China | 1  29  120 | 481  538-566  628-747 | MG604986 |
| GXBH1404 | China | 1  29  120 | 481  538-566  628-747 | MG604959 |
| GXYL1403e | China | 1  123 | 481  496-619 | MG604990 |
| GXNN1396 | China | 1  19  29 | 481  499-517  538-566 | MG604975 |
| GXNN1407a | China | 1  29  （1） | 481  538-566  （830） | MG604976 |
| GXBS1401a | China | 1  29  1 | 481  538-566  816 | MG604961 |
